# Predicting Risk of Mosquito-Borne Disease in Variable Environments

**DOI:** 10.1371/journal.pbio.0020390

**Published:** 2004-10-26

Malaria remains one of the greatest threats to global health, infecting more people than ever before. Confined mainly to the tropical areas of Africa, Asia, and Central America, malaria hits Africa the hardest; the poverty-stricken lands of sub-Saharan Africa account for 90% of malaria infections worldwide. Despite ongoing efforts to battle the disease—by controlling mosquito populations, reducing human contact, and developing drug prevention and treatment—the crisis continues to worsen.

The primary variables affecting risk of infection are the rate at which humans are bitten and the proportion of mosquitoes that are infectious. These two factors are often regarded as positively correlated, meaning that if the percentage of infectious mosquitoes increases, so will the human biting rate. But in a new study, David Smith, Jonathan Dushoff, and F. Ellis McKenzie challenge this assumption. Using a mathematical modeling approach to examine the relative contributions of the two factors across different landscapes and seasons, the authors show that the factors are not positively correlated. In fact, their calculations show that the rate humans are bitten and the proportion of infectious mosquitoes peak at different times and places.

Their modeling results suggest that the standard metric to estimate risk of infection—the number of times an infectious mosquito bites a person per day, called the entomological inoculation rate (EIR)—is flawed when variable conditions are taken into account. Using the average EIR to estimate average risk of infection in variable environments generates biased estimates because there is not a direct correlation between EIR and the proportion of humans who are infected.

The distribution of humans and suitable habitat for mosquito larvae varies across the landscape. And the density of mosquito populations varies seasonally, rising and falling with changes in rainfall, temperature, and humidity. Temporal and spatial variations in mosquito populations affect the rate humans get bitten, the number of infectious mosquitoes, and the risk of infection. To understand how these space- and time-induced variations in mosquito populations shape the epidemiology of human infection, Smith and colleagues developed a set of mathematical models that calculate the relative impact of different parameters, in order to determine which factors most influence where and when risk of infection is highest.

First, they evaluated what factors affect the primary components of the EIR: the human biting rate and the proportion of infectious mosquitoes. As expected, the model predicts that fluctuations in mosquito density influence the EIR by changing the human biting rate. As more people are bitten, more people become infected; consequently, more mosquitoes feed on infected humans and so become infectious. Only adult mosquitoes transmit infection, so as mosquito populations age, the proportion of infectious mosquitoes increases. During the dry season, few mosquitoes are born, and so while the human biting rate and EIR decline, the proportion of infectious mosquitoes increases.

Because mosquito populations are densest near breeding sites—where younger mosquitoes outnumber adults—the human biting rate and the number of bites by infectious mosquitoes per person per day reflect shifts in mosquito density, not in the proportion of infectious mosquitoes. The model predicts that human biting rate is highest shortly after mosquito population density peaks, typically either near breeding sites or where human density is highest. The proportion of infectious mosquitoes, on the other hand, reflect the age of the mosquito population: it peaks where older mosquitoes are found—farther from breeding sites—and when populations are declining.

By mapping larval habitats against the local risk of mosquito-borne infections, Smith and colleagues conclude, epidemiological models can be developed to predict risk for local populations. Their results make the case that mathematical models can help public health officials calculate risk of infectious diseases in heterogeneous environments—that is, real world conditions—when vector ecology and the parameters of transmission are well characterized. Any plan to prevent and control the spread of mosquito-born infections would clearly benefit from paying attention to mosquito demography and behavior.

